# Supplementary material for: Inharmonicity enhances brain signals of attentional capture and auditory stream segregation
Source: Commun Biol. 2025 Nov 17;8:1584. doi: 10.1038/s42003-025-08999-5 (PMC12623435; doi:10.1038/s42003-025-08999-5)
Supplement: Supplementary file 1 — Supplementary information [file 42003_2025_8999_MOESM1_ESM.pdf]

Supplementary information

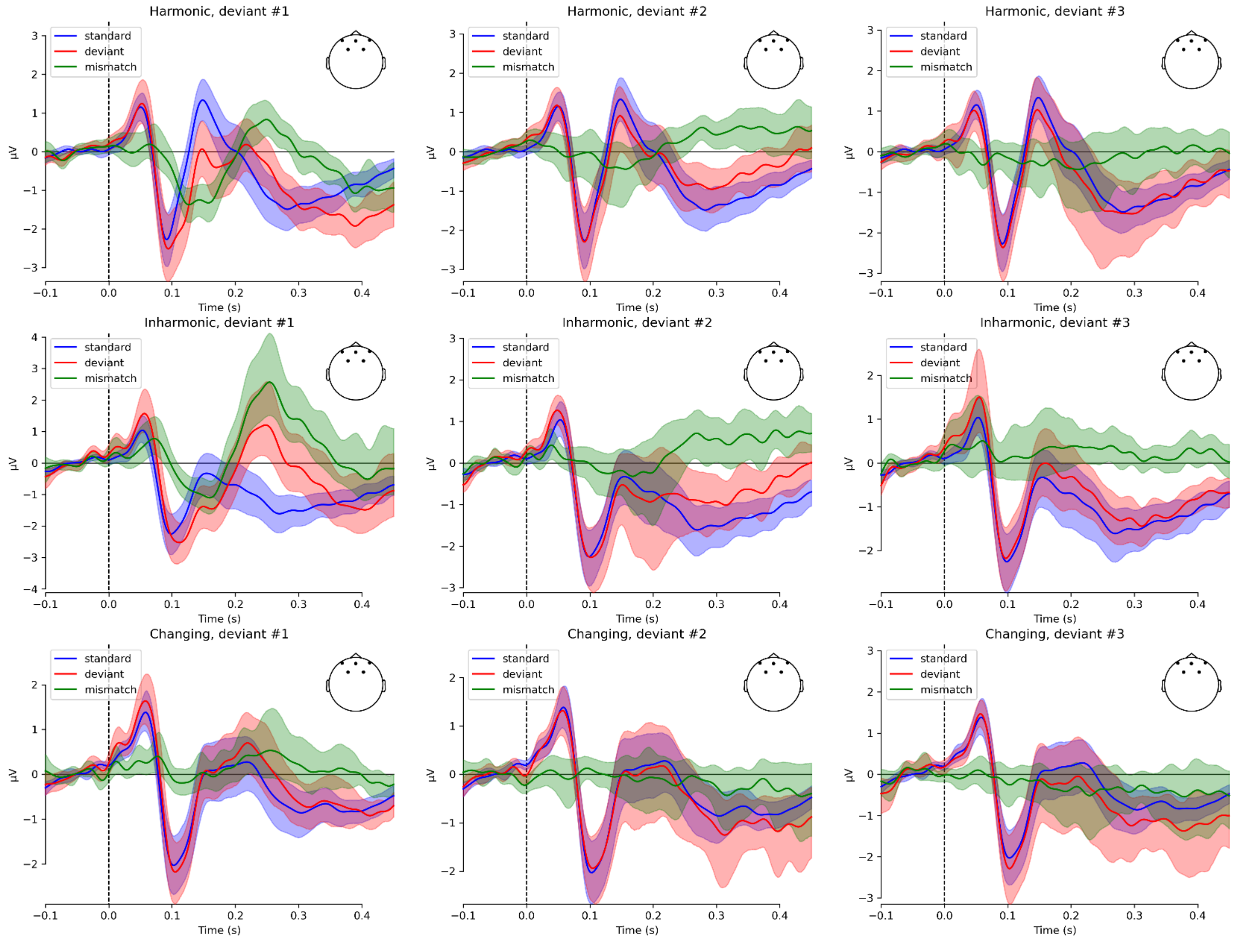

Supplementary Figure 1. Event-related potentials in three studied conditions for first-, second- and third-order deviants. Traces show grand-average responses to standards (blue), deviants (red) and the difference waves (green) for fronto-central channels (F3, Fz, F4, FC1, FC2). Color-shaded areas represent 95% confidence intervals.

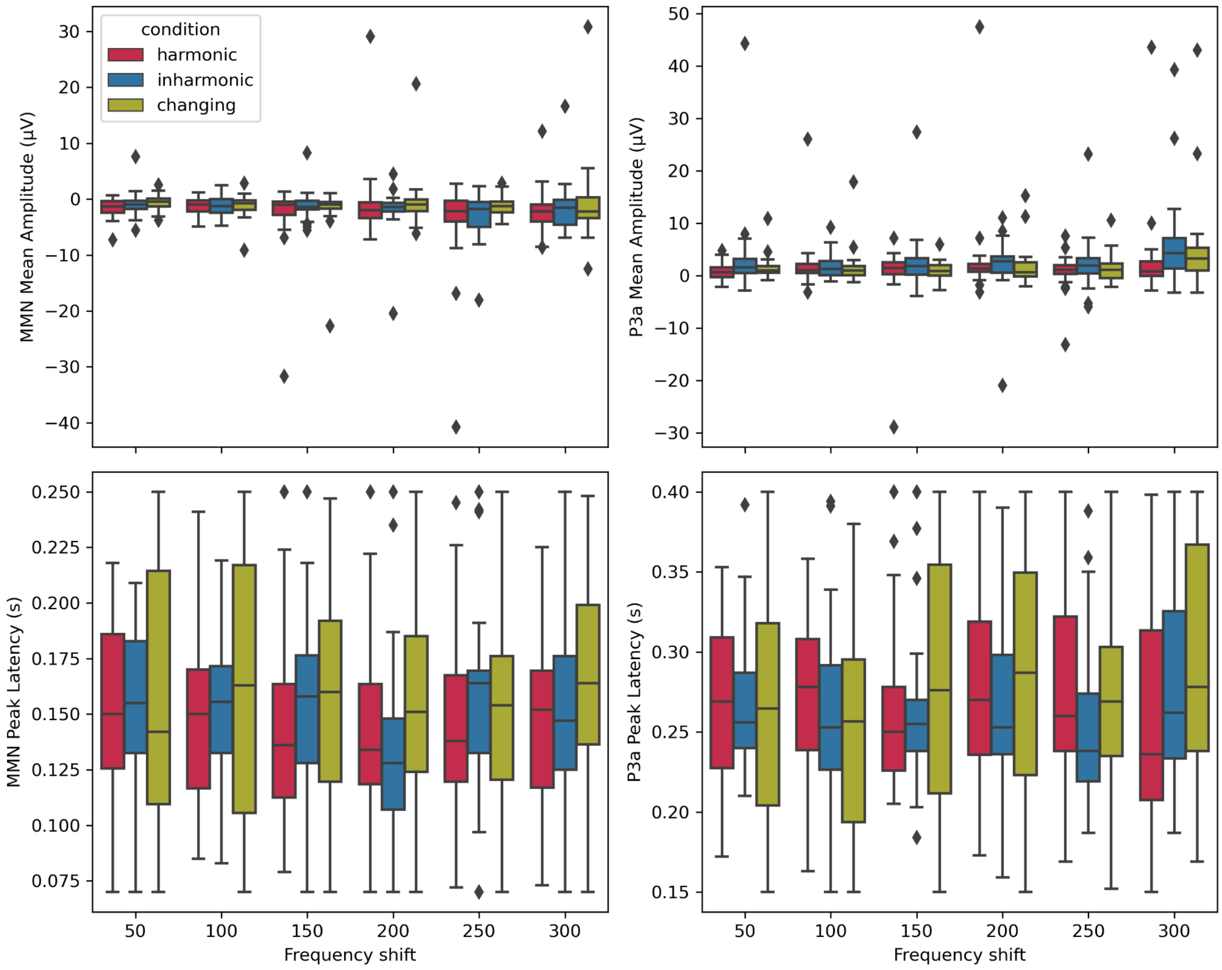

Supplementary Figure 2. Boxplots showing distributions of mean MMN (left panels) and P3a (right panels) mean amplitudes (top panels) and peak latencies (bottom panels) in the three conditions as a function of frequency shift.

Supplementary Table 1. Cluster-based analysis results for contrasts between standards and deviants. Table shows cluster p-values, start and end times (in seconds) and number of sensors in each cluster.

Condition	Cluster ID	P-value	T start (s)	T end (s)	No. sensors
harmonic	0	0.8989	-0.081	-0.062	6
	1	0.9597	-0.054	-0.038	4
	2	0.9997	0.03	0.039	1
	3	0.0001	0.087	0.219	26
	4	0.998	0.223	0.239	1
	5	0.9961	0.28	0.301	1
	6	0.0025	0.329	0.45	21
	7	0.9999	-0.037	-0.031	1
	8	0.9998	-0.005	0.003	1
	9	0.9055	0.087	0.134	1
	10	0.0763	0.216	0.286	16
	11	0.9989	0.313	0.326	1
inharmonic	0	0.9998	-0.086	-0.081	1
	1	0.9997	-0.054	-0.047	1
	2	0.0025	0.095	0.199	23
	3	0.0618	0.356	0.45	10
	4	0.4093	-0.035	-0.017	15
	5	0.999	-0.003	0.01	2
	6	0.5749	0.002	0.022	10
	7	0.1492	0.039	0.09	14
	8	0.0001	0.198	0.359	23
	9	0.9984	0.338	0.352	1
changing	0	1	-0.078	-0.073	1
	1	1	-0.071	-0.068	1
	2	0.9996	-0.05	-0.037	1
	3	1	-0.042	-0.039	1
	4	0.9848	0.034	0.062	1
	5	0.997	0.087	0.104	1
	6	0.7332	0.1	0.138	6
	7	1	0.162	0.165	1
	8	0.9824	0.371	0.39	3
	9	0.9887	0.402	0.419	3
	10	0.8501	0.432	0.45	6
	11	0.9996	-0.065	-0.054	2

Condition	Cluster ID	P-value	T start (s)	T end (s)	No. sensors
	12	0.9999	-0.023	-0.013	1
	13	0.9924	0.008	0.022	3
	14	1	0.043	0.044	1
	15	1	0.054	0.058	1
	16	1	0.064	0.072	1
	17	0.997	0.087	0.104	1
	18	0.7025	0.205	0.245	6
	19	0.904	0.252	0.29	2

Supplementary Table 2. Cluster-based permutations analysis for object-related negativity (ORN). Table shows cluster p-values, start and end times (in seconds) and number of sensors in each cluster.

Deviance	Condition	Cluster ID	P-value	T start (s)	T end (s)	Sensors
standard	harmonic-inharmonic	0	1	-0.1	-0.096	1
		1	1	-0.1	-0.094	1
		2	0.9993	-0.066	-0.059	2
		3	0.9803	0.004	0.021	2
		4	0.9944	0.033	0.051	1
		5	0.934	0.036	0.059	3
		6	0.0002	0.1	0.336	27
		7	0.3997	0.369	0.398	15
		8	0.4096	0.414	0.45	13
		9	0.9999	-0.019	-0.012	1
		10	0.9991	-0.019	-0.006	1
		11	0.9997	-0.017	-0.007	1
		12	0.2283	0.059	0.099	15
standard	harmonic-changing	0	1	-0.097	-0.095	1
		1	0.9357	-0.071	-0.056	5
		2	0.9995	-0.07	-0.059	1
		3	0.9999	-0.025	-0.02	2
		4	0.9979	0.053	0.068	1
		5	0.007	0.103	0.179	24
		6	0.9349	0.242	0.276	2
		7	0.7852	0.413	0.45	4
		8	0.9995	-0.07	-0.059	1
		9	0.9999	-0.043	-0.036	1
		10	0.3674	-0.024	0.023	14
		11	0.9165	0.023	0.034	8
		12	0.0189	0.053	0.101	28
		13	0.0006	0.208	0.396	25
standard	contrast	0	0.9994	0.173	0.183	1
		1	0.9886	0.251	0.273	1
		2	0.9997	0.429	0.438	1
		3	1	-0.1	-0.099	1
		4	1	-0.1	-0.098	1
		5	0.195	-0.002	0.04	19

Deviance	Condition	Cluster ID	P-value	T start (s)	T end (s)	Sensors
		6	0.0556	0.051	0.099	24
		7	0.0001	0.149	0.45	27
deviant	harmonic-inharmonic	0	0.9533	-0.1	-0.082	3
		1	0.9814	-0.1	-0.088	4
		2	0.9705	0.077	0.103	1
		3	0.0006	0.104	0.204	23
		4	1	-0.08	-0.076	1
		5	0.5343	-0.042	-0.011	15
		6	0.0236	0.05	0.12	24
		7	0.0275	0.223	0.342	14
		8	0.9939	0.265	0.291	1
		9	0.9991	0.296	0.314	1
		10	0.9996	0.341	0.356	1
		11	1	0.381	0.391	1
		12	0.9993	0.381	0.396	2
deviant	harmonic-changing	0	0.9998	-0.055	-0.044	1
		1	0.7981	0.08	0.14	1
		2	0.8054	0.123	0.147	5
		3	1	0.135	0.141	1
		4	0.9999	-0.015	-0.007	1
		5	0.9665	0.011	0.024	3
		6	0.0111	0.048	0.14	28
		7	0.9364	0.161	0.181	3
		8	0.0016	0.169	0.45	23
		9	1	0.44	0.444	1
deviant	contrast	0	1	-0.073	-0.068	1
		1	1	-0.03	-0.028	1
		2	1	0.042	0.044	1
		3	0.9614	0.046	0.058	5
		4	0.9978	0.099	0.117	1
		5	0.9873	0.121	0.148	1
		6	0.2476	0.237	0.319	16
		7	0.9958	0.331	0.352	1
		8	0.997	0.371	0.389	1
		9	1	-0.06	-0.054	2
		10	1	-0.053	-0.044	1
		11	1	0.022	0.029	1

Deviance	Condition	Cluster ID	P-value	T start (s)	T end (s)	Sensors
		12	0.0008	0.055	0.224	25
		13	0.1494	0.346	0.45	8
		14	0.9945	0.37	0.389	2

Supplementary Table 3. Cluster-based F-test for first-, second- and third-order deviants. Table shows cluster p-values, start and end times (in seconds) and number of sensors in each cluster.

Deviant	Cluster ID	P-value	T start (s)	T end (s)	No. sensors
First-order	0	1	-0.05	-0.047	1
	1	1	-0.035	-0.026	1
	2	1	-0.019	-0.015	1
	3	0.9994	-0.014	-0.007	2
	4	1	0.04	0.045	1
	5	1	0.048	0.056	1
	6	1	0.06	0.069	1
	7	0.0163	0.072	0.193	18
	8	0.9849	0.085	0.107	1
	9	1	0.193	0.204	1
	10	0.9935	0.201	0.215	2
	11	0.0075	0.211	0.345	19
	12	0.9989	0.264	0.278	1
	13	1	0.337	0.346	1
	14	0.1561	0.37	0.45	7
Second-order	0	0.9712	-0.1	-0.087	3
	1	0.9999	-0.092	-0.086	1
	2	0.5027	-0.022	0.01	9
	3	1	-0.009	-0.006	1
	4	0.9996	0.028	0.037	1
	5	0.9996	0.028	0.037	1
	6	0.0643	0.243	0.403	14
	7	0.9996	0.379	0.391	1
	8	0.5895	0.417	0.45	7
Third-order	0	0.9899	-0.062	-0.053	2
	1	1	-0.053	-0.048	1
	2	1	-0.047	-0.045	1
	3	0.9997	-0.01	-0.002	1
	4	0.9865	0.032	0.049	1
	5	1	0.071	0.075	1
	6	0.9993	0.102	0.112	1
	7	0.5878	0.114	0.148	4
	8	1	0.2	0.206	1
	9	0.9398	0.359	0.397	1
	10	0.9994	0.38	0.389	1

Supplementary Table 4. Model comparisons for the frequency shift analysis. The models include condition-only (m1), condition and frequency shift without interaction (m2), condition and frequency shift with interaction (m3). Model comparison is performed with a likelihood ratio chi squared test. Akaike Information Criteria (AIC) are presented for each model.

DV	Model	AIC	Statistic	Df	P-value
MMN mean amplitude	m1	3470.80			
	m2	3467.65	5.151	1	0.023
	m3	3470.09	1.556	2	0.459
MMN peak latency	m1	-2114.62			
	m2	-2112.99	0.369	1	0.543
	m3	-2109.27	0.276	2	0.871
P3 mean amplitude	m1	3712.23			
	m2	3702.90	11.333	1	0.001
	m3	3706.07	0.832	2	0.660
P3 peak latency	m1	-1705.49			
	m2	-1705.68	2.199	1	0.138
	m3	-1705.19	3.510	2	0.173

Supplementary Table 5. Post-hoc comparisons for P2 analysis of variance (model m2). T-tests with Tukey HSD correction for multiple comparisons.

Contrast	Estimate	Std. error	df	Statistic	P-value
harmonic deviant - inharmonic deviant	0.938	0.194	170	4.828	<.0001
harmonic deviant - changing deviant	-0.030	0.194	170	-0.154	1.0000
harmonic deviant - harmonic standard	-1.124	0.194	170	-5.787	<.0001
harmonic deviant - inharmonic standard	0.110	0.194	170	0.566	0.9931
harmonic deviant - changing standard	-0.128	0.194	170	-0.661	0.9859
inharmonic deviant - changing deviant	-0.967	0.194	170	-4.982	<.0001
inharmonic deviant - harmonic standard	-2.061	0.194	170	-10.615	<.0001
inharmonic deviant - inharmonic standard	-0.828	0.194	170	-4.262	0.0005
inharmonic deviant - changing standard	-1.066	0.194	170	-5.489	<.0001
changing deviant - harmonic standard	-1.094	0.194	170	-5.634	<.0001
changing deviant - inharmonic standard	0.140	0.194	170	0.720	0.9793
changing deviant - changing standard	-0.098	0.194	170	-0.507	0.9959
harmonic standard - inharmonic standard	1.234	0.194	170	6.353	<.0001
harmonic standard - changing standard	0.995	0.194	170	5.127	<.0001
inharmonic standard - changing standard	-0.238	0.194	170	-1.227	0.8233

P3 mean amplitude analysis - a possible outlier?

Visual inspection of the P3 mean amplitudes (Fig. 4) revealed a participant with P3 amplitudes higher than others. To verify the possibility that this outlier was driving the effect of harmonicity on P3 amplitudes, we conducted the same analysis with data from this participant excluded. The results showed that *m1* performs better than *m0*, (AIC_{m0} = 458.8, AIC_{m1} = 443.9, $Chi^2(2)$ = 18.80, $p < .0001$). Post-hoc comparisons revealed significant differences between harmonic and inharmonic conditions (contrast estimate = -1.36, SE = .36, t(68) = -3.85, p = .0008) as well as between inharmonic and changing conditions (contrast estimate = 1.45, SE = .36, t(68) = 4.07, *p* = .0004). No significant differences were found for the harmonic - changing contrast (contrast estimate = .08, SE = .36, t(68) = .23, *p* = .97). Taken together, these results suggest that the effect of harmonicity on P3 mean amplitude was not driven by an outlier observation.

Differences in topographies between ORN and MMN

To investigate if the topographies of ORN and MMN differed, we used a spatio-temporal cluster 1-sample t-test to compare MMN and ORN difference waves. We did this analysis for the time window of 100 - 200 ms where both components are negative. We contrasted MMNs in *harmonic* and *changing* conditions to ORNs for *inharmonic standards, inharmonic deviants and changing standards*. Since no MMN was detected for the *changing* condition, nor no ORN was detected for *changing deviants*, we did not include these contrasts in the analysis. Overall, six pairwise comparisons were made and none revealed significant clusters (all p > .05). These results do not support the notion that ORN and MMN have different topographical distributions. However, because we did not perform source analysis, this result does not preclude different neural sources of the components.

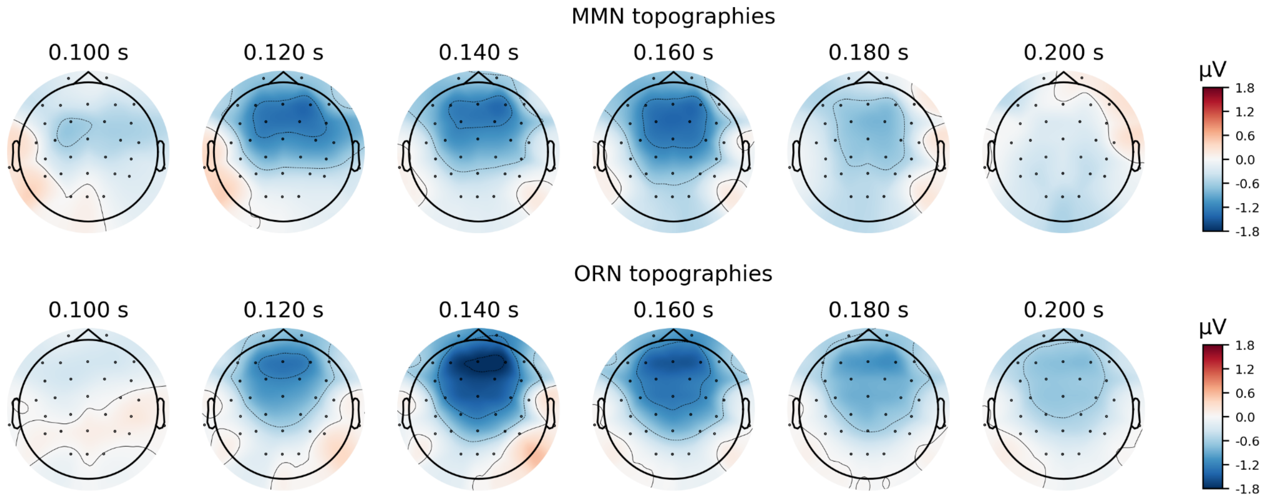

Supplementary Figure 3. Topographies of MMN and ORN responses. Upper panels show topographies of the *harmonic* MMN condition and lower panels show topographies of the *inharmonic standards* ORN.
