# Supplementary material for: Inharmonicity enhances brain signals of attentional capture and auditory stream segregation
Source: Commun Biol. 2025 Nov 17;8:1584. doi: 10.1038/s42003-025-08999-5 (PMC12623435; doi:10.1038/s42003-025-08999-5)
Supplement: Supplementary file 2 — Reporting Summary [file 42003_2025_8999_MOESM2_ESM.pdf]

nature portfolio

Corresponding author(s): Krzysztof Basiński

Last updated by author(s): Jul 18, 2025

Reporting Summary

Nature Portfolio wishes to improve the reproducibility of the work that we publish. This form provides structure for consistency and transparency in reporting. For further information on Nature Portfolio policies, see our Editorial Policies and the Editorial Policy Checklist.

Statistics

For all statistical analyses, confirm that the following items are present in the figure legend, table legend, main text, or Methods section.

n/a	Confirmed	
☐	☒	The exact sample size (*n*) for each experimental group/condition, given as a discrete number and unit of measurement
☐	☒	A statement on whether measurements were taken from distinct samples or whether the same sample was measured repeatedly
☐	☒	The statistical test(s) used AND whether they are one- or two-sided<br>*Only common tests should be described solely by name; describe more complex techniques in the Methods section.*
☐	☒	A description of all covariates tested
☐	☒	A description of any assumptions or corrections, such as tests of normality and adjustment for multiple comparisons
☐	☒	A full description of the statistical parameters including central tendency (e.g. means) or other basic estimates (e.g. regression coefficient) AND variation (e.g. standard deviation) or associated estimates of uncertainty (e.g. confidence intervals)
☐	☒	For null hypothesis testing, the test statistic (e.g. *F*, *t*, *r*) with confidence intervals, effect sizes, degrees of freedom and *P* value noted<br>*Give P values as exact values whenever suitable.*
☒	☐	For Bayesian analysis, information on the choice of priors and Markov chain Monte Carlo settings
☒	☐	For hierarchical and complex designs, identification of the appropriate level for tests and full reporting of outcomes
☐	☒	Estimates of effect sizes (e.g. Cohen's *d*, Pearson's *r*), indicating how they were calculated

Our web collection on statistics for biologists contains articles on many of the points above.

Software and code

Policy information about availability of computer code

Data collection	The stimuli were randomized and played back using PsychoPy (version 2022.1.3) for both EEG and behavioral experiments.
Data analysis	All EEG signal processing was performed in Python v.3.11 using MNE v.1.5 (Gramfort, 2013; Larson et al., 2024), Numpy v.1.24 (Harris et al., 2020) and Pandas v.2.1 (McKinney, 2010). Matplotlib v.3.7 was used for plotting (Hunter, 2007). Raw EEG data were high-pass filtered at 0.2 Hz, divided into epochs (from -100 ms to 450 ms) and entered into the autoreject algorithm v.0.4.2 (Jas et al., 2017). Linear mixed models were fitted in R v.4.4.1 (R Core Team, 2021) using lme4 v.1.1 (Bates et al., 2015). Model comparison was performed with a likelihood ratio Chi2 test. Post-hoc comparisons were performed by comparing the estimated marginal means calculated with emmeans v.1.10 (Lenth et al., 2021). P-values were corrected for multiple comparisons using the Tukey HSD method. Approximate entropies were calculated with AntroPy v.0.1.6 (Vallat, 2021). The raw data and code that was used to perform this experiment and analyze the results is available at https://doi.org/10.5281/zenodo.13939897 and https://doi.org/10.5281/zenodo.15236581 .

For manuscripts utilizing custom algorithms or software that are central to the research but not yet described in published literature, software must be made available to editors and reviewers. We strongly encourage code deposition in a community repository (e.g. GitHub). See the Nature Portfolio guidelines for submitting code & software for further information.

Data

Policy information about availability of data

All manuscripts must include a data availability statement. This statement should provide the following information, where applicable:

- Accession codes, unique identifiers, or web links for publicly available datasets
- A description of any restrictions on data availability
- For clinical datasets or third party data, please ensure that the statement adheres to our policy

The raw data and code that was used to perform this experiment and analyze the results is available at https://doi.org/10.5281/zenodo.13939897 and https://doi.org/10.5281/zenodo.15236581 .

Research involving human participants, their data, or biological material

Policy information about studies with human participants or human data. See also policy information about sex, gender (identity/presentation), and sexual orientation and race, ethnicity and racism.

Reporting on sex and gender	Gender information was collected via self-report from participants for demographic purposes only. The study was not concerned with any kind of gender or sex effect and this data was not used in any further analyses.
Reporting on race, ethnicity, or other socially relevant groupings	No data on race, ethnicity or other socially relevant groupings was collected in this study.
Population characteristics	See 'behavioural and social sciences study design'.
Recruitment	Participants were recruited using a university-hosted platform for volunteer recruitment to neuroscience experiments. The recruitment was automatic the the researchers could not influence it in any way other than by providing a study description for the participants. We do not expect that any biases arising from recruitment could realistically impact the data.
Ethics oversight	The study was approved by the department Institutional Review Board at Aarhus University (reference number DNC-IRB-2022-009) and followed the Declaration of Helsinki.

Note that full information on the approval of the study protocol must also be provided in the manuscript.

Field-specific reporting

Please select the one below that is the best fit for your research. If you are not sure, read the appropriate sections before making your selection.

☐ Life sciences ☒ Behavioural & social sciences ☐ Ecological, evolutionary & environmental sciences

For a reference copy of the document with all sections, see nature.com/documents/nr-reporting-summary-flat.pdf

Behavioural & social sciences study design

All studies must disclose on these points even when the disclosure is negative.

Study description	A quantitative experiment that involved recording electroencephalography while participants were exposed to acoustical stimuli.
Research sample	We recruited volunteers with no reported neurological or psychiatric illness, age between 18 and 45 years old, normal hearing, normal sight or corrected normal sight (e.g., contact lenses) and no use of medication that affects the central nervous system (e.g. opioids, pain medications).
Sampling strategy	Sample size has been calculated with a power analysis. The analysis showed that in order to achieve a statistical power of 0.8, at least N = 33 participants were required.
Data collection	Tones were presented using an oddball roving paradigm with an inter-stimulus interval of 600ms. Within each stimulus train, all standard tones had the same F0 and were followed by a train of stimuli with different F0 (chosen randomly from a 500 Hz - 800 Hz range in 50Hz intervals). In this paradigm, the first tone of a new stimulus train serves as the deviant stimulus and a potential source of mismatch negativity in context of prior stimuli. After a few repetitions, this deviant tone is established as the new standard until the next stimulus train is presented. The number of stimuli in a given train varied pseudo-randomly from 3 to 11, with 3 to 7 repetitions being four times more probable than 8 to 11. The entire procedure was divided into six blocks (two for each harmonicity condition), each 6 minutes long. Each block consisted of 600 sounds, with 98 deviants on average. The participants were asked not to move during the auditory stimulation, however there were short breaks between the blocks to provide rest and relaxation. All stimuli were administered passively and participants were watching a silent movie throughout the procedure. The stimuli were randomized and played back using PsychoPy (version 2022.1.3). Headphones (Beyerdynamic DT 770 PRO) were used for binaural sound presentation. The entire experiment lasted 80-90 minutes on average, including participant preparation and debriefing.

Timing	Data was collected between May and July 2023.
Data exclusions	We recruited 37 participants for the EEG experiment. One participant completed the study but was removed from analysis due to audio equipment failure during the procedure. Another participant was removed from analysis because of high levels of noise present in the data (over 60% of epochs excluded in the autoreject procedure). This noise likely resulted from a faulty electrode set.
Non-participation	No participant denied participation.
Randomization	The experiment was an entirely within-subject design.

Reporting for specific materials, systems and methods

We require information from authors about some types of materials, experimental systems and methods used in many studies. Here, indicate whether each material, system or method listed is relevant to your study. If you are not sure if a list item applies to your research, read the appropriate section before selecting a response.

Materials & experimental systems

n/a	Involved in the study
☒	☐ Antibodies
☒	☐ Eukaryotic cell lines
☒	☐ Palaeontology and archaeology
☒	☐ Animals and other organisms
☒	☐ Clinical data
☒	☐ Dual use research of concern
☒	☐ Plants

Methods

n/a	Involved in the study
☒	☐ ChIP-seq
☒	☐ Flow cytometry
☒	☐ MRI-based neuroimaging

Plants

Seed stocks	NA
Novel plant genotypes	NA
Authentication	NA
